# Managing intermittent preventive treatment of malaria in pregnancy challenges: an ethnographic study of two Ghanaian administrative regions

**DOI:** 10.1186/s12936-020-03422-2

**Published:** 2020-09-25

**Authors:** Matilda Aberese-Ako, Pascal Magnussen, Margaret Gyapong, Gifty D. Ampofo, Harry Tagbor

**Affiliations:** 1 University of Health and Allied Sciences, PMB 31, Ho, Volta Region Ghana; 2 Centre for Medical Parasitology, Faculty of Health and Medical Sciences, University of Copenhagen, Copenhagen, Denmark

## Abstract

**Background:**

Malaria in pregnancy (MiP) is an important public health problem across sub-Saharan Africa. The package of measures for its control in Ghana in the last 20 years include regular use of long-lasting insecticide-treated bed nets (LLINs), directly-observed administration (DOT) of intermittent preventive treatment with sulfadoxine-pyrimethamine (IPTp-SP) and prompt and effective case management of MiP. Unfortunately, Ghana like other sub-Saharan African countries did not achieve the reset Abuja targets of 100% of pregnant women having access to IPTp and 100% using LLINs by 2015.

**Methods:**

This ethnographic study explored how healthcare managers dealt with existing MiP policy implementation challenges and the consequences on IPTp-SP uptake and access to maternal healthcare. The study collected date using non-participant observations, conversations, in-depth interviews and case studies in eight health facilities and 12 communities for 12 months in two Administrative regions in Ghana.

**Results:**

Healthcare managers addressed frequent stock-outs of malaria programme drugs and supplies from the National Malaria Control Programme and delayed reimbursement from the NHIS, by instituting co-payment, rationing and prescribing drugs for women to buy from private pharmacies. This ensured that facilities had funds to pay creditors, purchase drugs and supplies for health service delivery. However, it affected their ability to enforce DOT and to monitor adherence to treatment. Women who could afford maternal healthcare and MiP services and those who had previously benefitted from such services were happy to access uninterrupted services. Women who could not maternal healthcare services resorted to visiting other sources of health care, delaying ANC and skipping scheduled ANC visits. Consequently, some clients did not receive the recommended 5 + doses of SP, others did not obtain LLINs early and some did not obtain treatment for MiP. Healthcare providers felt frustrated whenever they could not provide comprehensive care to women who could not afford comprehensive maternal and MiP care.

**Conclusion:**

For Ghana to achieve her goal of controlling MiP, the Ministry of Health and other supporting institutions need to ensure prompt reimbursement of funds, regular supply of programme drugs and medical supplies to public, faith-based and private health facilities.

## Background

In sub-Saharan Africa, where an estimated 25 million pregnant women are at risk of *Plasmodium falciparum* infection annually [1, 2], the interventions recommended by the World Health Organization (WHO) for malaria in pregnancy (MiP) have been included as a component of maternal healthcare [3–7]. Ghana adopted these interventions such as regular use of long-lasting insecticide-treated bed nets (LLINs), directly observed administration (DOT) of intermittent preventive treatment with sulfadoxine-pyrimethamine (IPTp-SP) and prompt and effective case management of MiP in 2003 [8–12]. Despite such interventions, Ghana like other sub-Saharan African countries, did not achieve the reset Abuja targets of 100% of pregnant women having access to IPTp and 100% of them using LLINs by 2015 [13].^1^ The sub-region has experienced challenges in the implementation of MiP interventions [13–20]. Health system challenges such as poor organization of health delivery, confusion over timing of each IPTp-SP dose, stock-outs, user fees and negative health worker attitudes have contributed to low utilization of MiP interventions [5, 21–28]. Socio-cultural challenges such as poverty, certain cultural practices, poor antenatal attendance and lack of knowledge on malaria and its effects on pregnancy have also affected uptake of MiP interventions [5, 15, 19, 22, 23, 26, 29–35].

Ghana has a relatively high percentage of women receiving antenatal care from skilled providers, which is reported at 97 percent in 2014 [36] and 98 percent in 2017 [37]. Forty-two percent out of the 2 percent who did not access ANC in 2017 reported that they failed to attend ANC, because they did not have money to pay for the service [37]. Sixty-four percent of women visited ANC for the first time in the first trimester and nine out of ten women visited ANC four or more times in 2017 [37]. Visiting ANC frequently has been proven to increase the likelihood of a woman completing five doses of IPTp-SP [38–41]. Yet, studies in Ghana have reported that there are still health system and socio-cultural challenges in the implementation of MiP interventions [14, 18, 21, 40–43]. Health system challenges include stock-outs of SP and LLINs, illegal charges and high cost of maternal healthcare [14, 21, 37, 44]. Socio-cultural challenges include negative attitudes in accessing IPTp-SP, poverty and some women attending ANC late and irregularly [18, 40, 41]. However, there is sparse literature on how healthcare managers deal with challenges in the implementation of MiP interventions in health facilities and the consequences on utilization of maternal healthcare and IPTp-SP services. This ethnographic study contributes to literature by exploring how healthcare managers and healthcare providers dealt with challenges in providing maternal healthcare and IPTp-SP services. Additionally, the study explored how such managerial actions influenced utilization of maternal healthcare and IPTp-SP services in two Administrative regions in Ghana.

### Context of MiP and maternal healthcare provision in Ghana

Ghana has integrated into Antenatal Care (ANC) service provision the issuing of IPTp-SP (3 tablets), which is given at 16th week of pregnancy every month till delivery, and issuing of LLINs to all ANC registrants. They are provided free of charge, alongside the free maternal healthcare policy intervention, which was introduced in Ghana in 2004 [10, 44, 45]. Fee-free maternal healthcare is factored into the National Health Insurance Scheme (NHIS), which pays health facilities for services and drugs provided to women who access ANC, delivery and post-natal care (PNC) [45]. The NHIS is currently operating a retrospective system—providers (accredited health facilities such as public, faith-based and private-for-profit) file claims for services provided and are reimbursed through the NHIS [46]. However, the National Malaria Control Programme (NMCP) procures LLINs, RDTs, ACT, SP and injectable artesunate for the Central and Regional Medical Stores and the two bodies supply public and faith-based facilities with the these medical products according to the needs of each facility [10]. To ensure continued distribution, the NMCP conducts periodic physical stock checks in central and regional medical stores [10]. Notwithstanding, the ‘Ghana Health Service and Teaching Hospitals Act (Act 525), which is the legislation that currently governs public sector service delivery, gives healthcare managers power to collect and retain 100% of user fees, referred to as internally generated funds (IGFs). Act 525 aims at ensuring that managers have decision-making power in order to be able to take prompt and independent decisions, to facilitate efficient and quality service delivery [47].

#### Analytical concepts: power and trust in health care

Power is central to decision making in both public and private institutions, as it determines everyday interactions between managers, workers and clients that access the institutions services. How power is exercised influences trust relations between managers and workers, as well as workers and the clients that utilize the institution’s services. This study uses concepts of power and trust to discuss how the decisions that managers make can affect health care delivery and utilization.

Mintzberg [48], defines power in organizations as “…*the capacity to effect (or affect) organizational outcomes*.” Mintzberg’s definition of power is appropriate for this study, because it enables an interrogation of power in both public and private institutions in developing countries such Ghana [49–52]. Healthcare managers’ sources of power include authority vested in the office of the manager, networks and access to resources [48, 53, 54]. While Act 525 devolves vertical power to healthcare managers in Ghana, in order to facilitate quality health care provision [47], questions of whether managers have actual resource power and how they make decisions in relation to resources is central to the functioning of the Ghanaian health care system [52, 55]. Pfeffer [53, 56], argues that there is excessive centralization in both private and public organizations, which leaves managers with very little decision making power. Yet, managers in public institutions such as health care institutions are held accountable for organizational outcomes and are expected to be responsive to the varied demands of stakeholders such as healthcare workers, external actors including governmental bodies, donors and clients who patronize health care. They are expected to exercise power in the implementation of policies that contribute to the achievement of organizational goals [52, 56]. How healthcare managers exercise power in everyday decisions in health facilities shapes how health care is provided and determine the quality of care that clients receive [57–60].

When managers exercise power positively such as providing resources for work, workers develop trust in managers [61]. Health workers are then able to provide quality care, which helps clients to develop trust in healthcare providers and the health care system [58, 60]. Where managers exercise power negatively such as not providing work resources, workers lose trust in managers. When workers lose trust in managers they exhibit negative attitudes towards clients, which can result in clients distrusting the health care system [57, 61]. Trust has been defined as “…*the optimistic acceptance of a vulnerable situation in which the trustor believes the trustee will care for the trustor’s interests*” [62]. Effective health care requires a social relation of trust between health providers and clients. Trust affects the attitudes and behaviors of clients in decisions on health care [62–65]. When clients perceive negative exercise of power in health facilities, they respond by finding alternative sources of health care, refusing to comply with scheduled visits and failing to adhere to treatment. However, when power is exercised positively, clients develop trust in health providers, so they patronize health services and adhere to treatment. Health manager—worker trust relations has equally been explored [66]. When healthcare managers provide resources for healthcare provision, health workers develop trust in managers, as they are able to exercise power positively, by providing quality health care. When healthcare managers fail to meet workers’ work needs, they lose trust in healthcare managers and become demotivated in the provision of health care [61, 66, 67]. Demotivation can affect health workers ability to provide quality health care to clients [61].

## Methods

### Study design

The study design was ethnographic. It employed non-participant observations, conversations^2^ and in depth interviews (IDIs) to obtain data from healthcare managers, health workers, NHIS personnel, pregnant women and a cross section of community members. The study team collected data from April 2018 to March 2019, while follow-up interviews and conversations were conducted in September 2019. The choice of ethnography enabled the study to achieve its objective of exploring behavioural factors influencing access to IPTp-SP from different perspectives such as healthcare managers, health providers and pregnant women. Using ethnography to explore specific, as well as complex phenomena in the health care environment is common and relevant to understanding behavioural factors influencing health care provision and access to health care [68, 69]. Various scholars have conducted ethnographic studies in different locations in hospitals such as in wards, on health care interventions and health care policy issues [70–73]. Such studies have contributed to understanding behavioural factors influencing health care provision and utilization [74].

The research team comprised of a post-doctoral researcher (MA) and eight research assistants (RAs).^3^ MA trained the RAs and supervised data collection. Each RA was assigned to a facility and a community for data collection. The team conducted non-participant observations intermittently in the 8 facilities and 8 communities.

### Selection of research area

The study was conducted in five districts,^4^ three in the Ashanti and two in the Volta regions of Ghana. Eight health facilities (five government and three faith-based) and 8 communities were chosen for the study. Details of the selection process are indicated in subsequent paragraphs.

Ashanti region was selected to represent the middle belt of the country, while Volta region was selected to represent the southernmost belt of Ghana. The two regions are linguistically different, Twi is spoken in the Ashanti region and Ewe is spoken in the Volta region. Ashanti Region reported the second highest percentage (98.8%) of women receiving ANC care from skilled providers in 2014, while the Volta region reported the second lowest percentage (93.9%) of women receiving ANC from skilled providers [37].

The district hospitals in the five districts qualified automatically to participate in the study. Also, interactions and interviews with pregnant women in some of the study communities revealed that they preferred to visit particular health facilities for ANC services. Three of such facilities, which are faith-based were included in the study. Thus, a total of 8 health facilities were selected for the study. Some women preferred the three facilities (2 in the Ashanti region and 1 in the Volta region), because they were closer to their communities than the district hospitals. The women’s assertion of nearness to facilities was further confirmed by transect walk in all the communities to confirm the location of health facilities.

The study team visited the study health facilities and went through ANC case records. The team counted the total number of MiP cases documented in the ANC records for each community that accessed ANC in a study facility, from January 2015 to March 2018. The community with the highest recorded number of pregnant women who were diagnosed of malaria in each facility was chosen for the study. Four study communities in the Ashanti and 4 in the Volta Region were selected for the study (Table 1). Hospital records were used as a criteria to select study communities, because it increased the team’s chances of including pregnant women who had experienced malaria during pregnancy in communities that had a history of MiP case. Two other reasons for using hospital records to guide the selection of study communities were: (1) earlier literature suggested that Ghana had high ANC attendance rate and high IPTp-SP + 1 uptake [36, 37]; (2) health providers in study facilities confirmed that malaria cases among pregnant women had reduced drastically compared to the general population, due to an increase in uptake of IPTp-SP.

**Table 1:** Study health facilities and communities in the Ashanti and Volta Regions with pseudonyms

Type of study site	Region	
	Ashanti* No.	Volta^#^ No
Hospital(s)	3	2
Health Centre(s)	1	2
Ownership of facility		
Government owned	2	3
Mission owned	2	1
Communities	4	4

*Study facilities in the Ashanti region have been given the pseudonyms: ASF01, ASF02, ASF03 and ASF04. Study communities in the Ashanti region have been given pseudonyms: ASC01, ASC02, ASC03, ASC04

#Study facilities in the Volta region have been given the pseudonyms VRF01, VRF02, VRF03, VRF04. Study communities in the Volta Region have been given pseudonyms: VRC01, VRC02, VRC01, VRC01

### Community entry

The research team held a sensitization workshop for the Volta Regional health directorate and the district health management teams (DHMT) of participating districts in the Volta region. The regional health director and the DHMT directors approved the study. A sensitization workshop was not carried out in the Ashanti region, because some of the team members in this current study had conducted community entry activities in the region and in the study districts in earlier studies. However, the team sent letters to all the participating DMHTs in the Ashanti and Volta regions, to seek permission to conduct the study. The DHMTs approved the study by writing letters to that effect. Copies of the letters approving the study were sent to the heads of the study health facilities in each study district and leaders of the study communities. The study team met assembly members, chiefs, queen mothers and elders in each study community, to inform them about the study and to seek permission to conduct the study in their communities.

### Selection of study participants

A research assistant was assigned to a health facility to conduct non-participant observation, to conduct conversations and IDIs with health workers and women who were attending ANC. The RAs first immersed themselves in the study facilities and the communities [75]. The RAs took the following steps: familiarized themselves with study facilities and communities; developed trusting relations with potential study participants, identified places that pregnant women liked to visit and carried out transect walk. The approach provided MA and the RAs information on where and how to recruit study participants using convenience and snowball sampling methods.

The RAs used convenience sampling to select pregnant women attending ANC. They approached women who were attending ANC and those who confirmed that they were residing in a study community were invited to participate in IDIs. The RA explained the study to the women and those who were interested offered their contact addresses. The RAs later visited them at home to obtain written consent and to conduct IDIs. Pregnant women were also recruited from study communities, using snowball method. The first woman who was approached in the community or at the health facility helped the RA to identify other pregnant women in her community.

Convenience sampling was used to select women who participated in conversations. RAs approached women who were visiting ANC and those who were interested in conversing with RAs gave verbal consent. The RAs accompanied them while they accessed different ANC services. The purpose was to learn and to understand women’s experiences in going through the ANC process.

Information that the study team gathered from immersing themselves in the study field and maps that were sketched from transect walk in the 8 study communities, guided the team in selecting case studies. Case studies were purposively selected among women who attended ANC every month, those who attended ANC irregularly, those who skipped ANC appointments and those who were being treated for malaria.

Healthcare providers (nurses and midwives) who had been working in the ANC for at least 1 year, were purposively selected to participate in the study. The choice of health providers who had worked over a year, was to ensure that health providers who were selected had adequate knowledge on the subject of interest. Additionally, healthcare managers (senior medical officers and physician assistants in-charge of hospitals and health centres and healthcare administrators), ANC managers (senior midwives in-charge of the ANC), and laboratory officials were purposively selected to participate in the study (Table 1).

### Data collection techniques

RAs visited case studies at home, to observe whether they used LLINs, whether they attended ANC regularly and whether they took their medications. The RAs conducted IDIs with the women and community members using Ewe language in the Volta region and Twi language in the Ashanti region. The IDIs centred on knowledge, attitudes, beliefs and practices on malaria in pregnancy interventions and socio-cultural practices. The IDIs that were conducted with healthcare providers centred on maternal and MiP service provision, challenges and facilitators. The RAs interviewed or talked to procurement officers and laboratory personnel, to clarify issues on payment processes and stock-outs of drugs and other medical products. ANC unit managers commonly referred to as in-charge and healthcare managers were interviewed, to help understand managerial and administrative practices. The study team talked to some of the officials of the national health insurance scheme (NHIS), to clarify and verify issues raised by healthcare managers such as NHIS policies and delays in reimbursement for some ANC and MiP services (Table 2). English language was used in conducting IDIs and conversations with healthcare workers and NHIS officials, because it is the official language of Ghana (see Additional files 1, 2, 3, 4, 5, and 6).

**Table 2:** Data collection methods and categories of respondents

Region	Category of respondents	IDIs	Conversations	Case studies
Ashanti				
	Facility managers	4	0	0
	ANC managers	4	4	
	Health care providers	11	20	0
	Pregnant women	30	25	4
	Procurement officers	1	2	0
	Laboratory officials	0	6	0
	Pharmacy officials	0	4	0
	DHD Officials	0	2	0
		0	1	0
	Total	49	56	4
Volta Region				
	Facility Managers	4	0	0
	ANC managers	6	4	
	Health Care providers	12	20	0
	Pregnant women	40	32	8
	Laboratory officials	0	2	0
	Pharmacy Officials	0	4	0
	DHD Officials	0	2	0
	Total	74	64	8
	NHIS officials	0	2	0

Observations were conducted in 8 health facilities and 8 communities in the Ashanti and Volta regions for 12 months

RAs conducted transect walk in study communities to identify key places, settlement patterns and physical access to health care facilities and other sources of health care. The RAs sketched maps from the information obtained from the transect walk, which guided the study in choosing three additional facilities, locating pregnant women and case studies.

### Data analysis

Interviews were recorded using digital recorders and transcribed verbatim to preserve interviewees’ original messages. Interviews in Ewe and Twi were transcribed into English to enable easy analysis and comparison. This study was conducted as part of a larger study on parasitic infections during pregnancy and community interventions programme in Ghana. The study set out to explore socio-cultural and community factors influencing utilization of MiP interventions in Ghanaian communities. Aspects of the study has been reported elsewhere [14]. However, initial analysis of the data revealed that multiple community and health system factors influenced utilization of MiP interventions. This informed the team to return to the study health facilities to explore and understand health system factors influencing utilization of MiP interventions.

Grounded theory approach was used in data analysis [76]. The first set of data (interviews, observation notes and conversations) was triangulated and an iterative approach was used to code the data. MA and ED (ED is a qualitative data analysis expert, who was hired to assist in coding) developed a codebook and used it to code the data with the assistance of qualitative analysis software, Nvivo Version 11. The codes generated by MA and ED were compared, agreed and merged. Key informants such as healthcare managers and health workers were visited for further interviews in pursuance of emerging themes which included the following: (1) why and how managers made certain decisions on maternal healthcare; (2) why ANC providers who claimed they loved their jobs felt frustrated in providing maternal healthcare; and (3) why women did not trust the health care system and were skipping appointments. Coding, analysis and data collection went on until saturation was attained. Saturation was attained when no new information on the major themes were obtained, which is in line with Charmaz’s [76] recommendation. The results of this manuscript are based on major themes that emerged from the analysis.

### Ethical issues

Ethical clearance was obtained from the University of Health and Allied Sciences’ Research Ethics Committee [UHAS-REC/A.I Ul 17-18]. The team obtained written consent from study participants who participated in IDIs and verbal consent from those who participated in conversations.^5^ To protect informants’ identity, besides actual country and region names, individuals and facilities’ names used in this article are pseudonyms.

## Results

### How healthcare managers dealt with MiP implementation challenges

Health facilities faced challenges such as frequent stock-outs of malaria programme drugs and supplies from the National Malaria Control Programme (NMCP), delayed reimbursement of funds from the NHIS and reduction in the supply of medical products from the Ministry of Health (MOH). Healthcare managers used their power under Act 525 to address the challenges by adopting the following strategies: (1) instituting co-payment for drugs and services that were previously free such as the SP drug and malaria test for pregnant women; (2) rationing SP and (3) prescribing drugs, which were previously issued under the NHIS for clients to buy from the open market. Healthcare managers explained that they made such decisions in order to ensure that they always had funds to purchase essential medical supplies and drugs, as well as to pay casual labourers and suppliers, in order to sustain health service provision. Some facilities neither sold drugs to clients nor charged fees for some of the services that they provided to clients. Sometimes such facilities experienced stock-outs of critical drugs and supplies that workers needed to enable them to provide quality health care.

Consequently, some women could not afford maternal healthcare, so they visited multiple sources for health care services such as ANC, herbalists and prayer camps. Others took herbs, skipped scheduled ANC visits or started ANC late. Such actions led to some women initiating SP late, taking SP irregularly or adhering poorly to malaria treatment. So, women’s expectations of enjoying comprehensive maternal healthcare was not met and some lost trust in the health care system. Nevertheless, clients who could afford maternal healthcare appreciated the seamless flow of services. Healthcare providers in facilities that experienced stock-outs felt frustrated, demotivated and lost trust in healthcare managers. The different issues are further discussed below.

### How health facilities dealt with challenges in MiP policy implementation arrangement

Healthcare managers and health workers reported in interviews that the National Malaria Control Programme (NMCP) is responsible for providing both government and faith-based facilities with malaria programme drugs and supplies such as SP, malaria test kits (RDT) and LLINs, through the Central Medical Stores (CMS) and the district health directorate (ASF01, IDI, Healthcare Manager; VRF02, IDI, Healthcare Manager). Nonetheless, they indicated that sometimes facilities experienced stock-out of SP and RDTs, because the CMS could not supply them with SP all the time and it had stopped suppling them with RDTs.

Healthcare Managers in ASF02, ASF03 and ASF04 in the Ashanti region reported that they decided to use internally generated funds (IGF) to procure SP from private manufacturing companies, whenever there was stock-out. To recoup the money, they sold SP (which was previously free) to women at a reduced price, which was dubbed ‘top up’. They added that facilities offered SP free to women whenever the CMS supplied facilities with SP. An ANC manager stated:

“*The district health directorate used to supply us with the SP. The district health directorate no longer supplies us with the SP. Sometimes, we go to the district pharmacy for the SP, but it is not forth coming, so I bought this one (pointing to a box containing SP on the table in the ANC consulting room) from outside market to avert stock out of the SP.”* (ASF02, IDI, ANC manager)

All the study facilities in the Volta Region and one facility in the Ashanti region did not buy SP from the open market, so they run out of stock whenever they were not supplied SP, as a manager explained:

“*For medicines, those that we are supposed to get from the open market are always available, but the programme drugs like the SP…if it is not available at the District Medical Store or Regional Medical store, we cannot get it from anywhere. Sometimes it [unavailability of SP] affects our clients*.” (ASF01, IDI, Healthcare manager 01).

Some of the facilities that experienced stock-out of SP issued prescriptions to women who attended ANC to buy SP from private pharmacies. Such facilities could not enforce DOT on such occasions, since clients did not return to the facilities to take the purchased SP in the presence of healthcare providers. However, some of the healthcare providers in ASF02 and VRF04 compelled women to return to the ANC to take the purchased SP under DOT. Healthcare providers succeeded in doing that by seizing their clients’ maternity records booklets, which were given back to them, after they had returned to take the purchased SP under DOT.

Whenever VRF02 experienced stock-out of SP they asked clients to return to the facility at a later date, when the facility would have replenished its stock. Some clients returned after a week or more to take SP under DOT (VRF02, Observation notes, 02/07/2019). VRF02 also borrowed SP from other government health facilities whenever they run out of stock (VRF02, IDI, Healthcare manager). One of the strategies that ASF04 adopted was to ration SP to clients, as a way of addressing shortage of SP. Thus, some women who attended ANC early and regularly, ended up taking 3 doses of SP instead of 5 or more.

Some facilities used part of their IGF to buy RDT kits and reagents, so they charged fees in order to recoup the money. Thus the policy of fee-free testing for malaria in pregnancy service was changed to fee-paying.

Healthcare managers in three study health facilities in the Ashanti Region explained that they had to buy anti-malarial drugs from the open market to sell to clients. However, quinine was administered at no cost to the women. Nonetheless, some of the facilities in the Volta Region such as VRF04 gave artesunate-amodiaquine drug free to pregnant women, who were diagnosed of malaria.

### Delayed reimbursement from the National Health Insurance Scheme and hidden cost of MiP and maternal healthcare impacted on access to MiP interventions

Most of the fees were paid at the pharmacy and receipts were issued to the clients. The institution of fees contributed to increasing cost of ANC services. Healthcare managers instituted fees for some of the medical products and services that were offered to clients. They explained that they took such a decision because, the NHIS usually delayed for over 6 months before reimbursing the facilities for the cost of services provided. An official of the NHIS confirmed that some health facilities received reimbursement for services that they provided in 2018 in 2019, which was quite late (Conversation with an NHIS official, 11/03/2019). Yet, facilities had to buy drugs and equipment, pay contract staff and suppliers in order to keep their facilities functioning. So managers in most of the facilities used their power to institute various forms of co-payment for services that were previously free. For instance in three study facilities in the Ashanti region women who were NHIS subscribers paid half the price of routine drugs such as folic acid, ascorbic acid, fersolate and vitamin B complex, while uninsured women paid the full cost (ASF02, Observation notes, 27/08/2018; ASF04, Observation notes, 16/08/2018; ASF03, IDI, Healthcare Manager). However, all the four facilities in the Volta Region gave routine drugs to ANC clients for free.

Women had to undergo urine in pregnancy test (UPT), which costs GH₵5 (1$), before they could be enrolled onto the NHIS to access fee-free maternal healthcare and malaria related services. NHIS reimburses facilities for services provided to individuals who are registered subscribers. So, since majority of the women, who attended ANC for the first time were not insured, the facilities did not obtain reimbursement from the NHIS. The facilities passed the cost to the clients, as they could not afford to use IGF to offer women free UPT service. Health providers in interviews explained that a second reason for mandatory pregnancy test was to prevent non-pregnant women from exploiting fee-free maternal healthcare service (ASF01, Conversation, Midwife, 19/11/2018; ASF01, IDI, Healthcare Manager).

In four facilities ANC clients paid 5GHS (1$) for a maternity record booklet. Healthcare managers explained that the facilities offered free maternity booklets to women when MOH supplied them with booklets. However, the MOH had not supplied them with booklets in the last 6 months. Consequently, the facilities decided to use some of their IGF to print maternity booklets, so they passed the cost to the women. They further argued that the booklets were relevant, because they ensured that individual maternity records were well documented, to facilitate provision of quality maternal healthcare (VRF03, conversation, Healthcare Manager, 30/11/2018; ASF01; IDI, Healthcare Manager 02).

Women who were taking SP for the first time in their current pregnancy, were required to undergo a glucose-6-phosphate dehydrogenase (G6PD) test (the test assists in determining whether a client could be put on SP). The cost of the test is between GH₵15.00 and GH₵20.00 ($2.8–$3.72) in four facilities. Three facilities explained that women had to pay, because the test is not part of the fee-free NHIS package that pregnant women are supposed to enjoy. However, in VRF03 insured clients paid a top-up of GH₵5 (1$) for the test and uninsured clients paid GH₵15 ($3). A healthcare manager explained that the facility introduced payment for the service in 2019, because the government had stopped reimbursing the facility for G6PD test in 2018 (VRF03, IDI, Healthcare Manager).

In some facilities before women were given their first dose of SP, they were required to undertake a microscopic examination of blood for malaria parasites (utilizing blood films (BFF test), which costs GH₵5 (1$).

### Perceived negative influence of decisions taken by health facilities in addressing maternal healthcare and MiP services on women’s health-seeking behaviour

Some women could not access comprehensive maternal healthcare, because they could not afford to pay for drugs, laboratory and ANC services. Observations in VRF04 and ASF01 illustrate challenges that ANC clients experienced in paying for maternal and MiP services. Client Ajo (all clients are referred to by pseudonyms), a pregnant woman was referred to the laboratory for haemoglobin (Hb) test. However, she explained to the midwife that she was reluctant to take the test, because her husband did not give her enough money for the ANC visit (VRF04, observation notes, 30/07/2018). Client Cynthia, who was 4 months pregnant, complained of losing appetite, feeling weak and dizzy for a number of days. So, a midwife referred her to the resident obstetrician-gynecologist, on suspicion of malaria. Client Cynthia was seen sneaking out of the facility. When she was confronted by an RA, she explained that she could not afford the cost of laboratory test. Also, if the test confirmed that she had malaria, she will not be able to afford the cost of treatment, so she was going home to seek alternative treatment (ASF01, observation notes, 20/08/2018).

Some ANC attendees skipped scheduled ANC appointments, because they could not afford comprehensive ANC care. The research team visited two case studies, a 17-year old adolescent and a 27-year old woman in VRC02. Both of them were NHIS subscribers and their houses were a walking distance to VRF02. Yet, both of them had missed their scheduled ANC visit for the month of July 2018, because they could not afford the cost of ANC (VRC02, conversation with two case studies, 19/07/2018).

Other women who could not afford the cost of comprehensive ANC service sometimes visited herbalists and prayer camps, stayed at home or prayed for divine intervention. A respondent whose house was very close to ASF02, but had stopped attending ANC stated:

“*At the early stage of my pregnancy, I felt weak and I was unable to do anything that is why I am no longer working. When I asked my husband to give me money to go to hospital, he told me he didn’t have money… So I was using my own money. I have been there [ASF02] 3 times and now I don’t have money to go again. …I don’t go to hospital and I have been using local medicine. I pray to God to give me life, strength and protect my child and myself, so that nothing bad happens to us*.” (ASC02, IDI, Pregnant Woman 02).

Some women were happy to take the prescription forms that the health providers and pharmacists offered them, whenever the facilities did not have a recommended drug in stock. This was because the health providers could not compel them to purchase the prescribed drugs, once they left the hospital. So, some did not buy the prescribed drugs and returned to the facility later with the same complaints: “… *those who want to buy will buy. Others will also leave the prescriptions in their ANC booklet and come with the same complaints on their next visit.*” (VRF02, IDI, ANC Manager).

In order to avoid paying fees at the ANC, some women waited till they were six on more months pregnant before attending ANC. Consequently, they initiated IPTp-SP late and did not complete the recommended five doses of SP (ASF01, IDI, Health worker01). Some women were discouraged from starting ANC early, by female relatives and friends, who shared their experiences of paying for ANC services. An IDI with a 16 year old adolescent, who was 7 months pregnant, but had never attended ANC revealed:

- Interviewer: “Why don’t you go for ANC?”
- Adolescent: “I don’t have money.”

Interviewer: “You said you haven’t started ANC, so how did you know you pay money there?”

- Adolescent: “One of my sisters told me.”
- Interviewer: “So if you had the money, would you have gone?”
- Adolescent: “Yes, as for the ANC it is good. When you go they give drugs.”
- (ASC03, IDI, Pregnant Woman 12)

Several women who could not afford maternal healthcare services and those who were given prescriptions to purchase certain drugs from the open market, lost trust in the health care system. They perceived that the system was not responsive to their health needs. Some women visited other health facilities, when they were told that more drugs were being provided in those facilities. Others visited prayer camps and herbal centres, while some women engaged in self-medication. A 17-year old adolescent, who was 6 months pregnant, skipped her last scheduled ANC visit, because she did not have money. However, she attended prayer sessions at a prayer camp every Thursday. She indicated that she trusted the prayer camp more than the health facility as follows:

*“The hospital will only see the physical and the best that they can do for you is to prescribe drugs for you to go and buy and take. But the prophet can see both physical and spiritual. After consultation, he would give you herbs to go and take and after three days you will be well. He can also foresee and avert any misfortune that can happen in the course of the pregnancy. So for me I trust the prophet more.”* (VRC02, Conversation, Case Study, 19/09/2018)

### Perceived positive aspects of decisions taken to facilitate MiP and maternal healthcare delivery

It was observed that women who were gainfully employed and women whose husbands encouraged them or gave them financial support were able to afford the cost of ANC. Others received financial support and encouragement from their mothers, mothers-in-law, sisters and friends to attend ANC services. A team member interacted with a woman at the ANC, who stated her reason for attending ANC:

“*My friend who influenced me to come to ANC said something about the drug [SP] at the time she [her friend] was advising me to come for ANC.”* (ASF02, Observation notes, 23/08/2018).

Some women attended ANC regularly, because they had built trust in the health care system, resulting from having experienced positive effects of utilizing health care in previous pregnancies. Others voluntarily accessed health care and did not complain about the cost of MiP and other maternal healthcare services. Other women were influenced to attend ANC regularly by multiple factors such as encouragement from husbands and healthcare providers and to prevent complications during childbirth. An interview with a pregnant woman illustrates that multiple factors influenced decision to utilize ANC services:

- Respondent: “I started ANC in the fourth month, because I wanted to prevent any complications.”
- Interviewer: “What are the factors that influenced you to attend ANC?”
- Respondent: “I decided to start attending ANC by my own will, but not for economic, or distance factors.”
- Interviewer: “Who decides when you should start attending ANC?”
- Respondent: “My husband.”
- Interviewer: “Who prompts you to go for ANC?”

Respondent: “My husband, but when we go for ANC, I am told [by the health provider] the next time I should come, so when the time comes, I go.” (VRC04, IDI, Pregnant Woman 05)

An initiative known as “last mile” was introduced in the second quarter of 2019. Health facilities requisition for medical commodities to last for 3 months and the CMS sends them to the facilities every 2 months. The initiative ensures that all facilities including those that are located in remote areas have unlimited access to medical commodities including SP and other malaria drugs. However, facilities still experience shortage of medical consumables such as gloves, gauze among others, because those items are not supplied to them under the initiative. (VRF02, IDI, Healthcare Manager; VRF04, IDI, Healthcare Manager).

### Effects of managers decisions on maternal health and MiP services provision

Healthcare managers perceived the decision to pass on the cost of maternal healthcare and MiP services and drugs to clients as beneficial. The money generated was used to buy drugs, medical supplies and consumables for service delivery.

Some of the midwives reported in interviews that pregnant women who attended ANC were always reluctant to undertake laboratory tests, because of the fees involved. On one occasion a client was reluctant to undergo hb test, because of the cost involved, so a health provider stated: “…*the pregnant women in this town* [VRCy04] *… do not like paying at all for health care services.*” (VRF04, observation notes, 30/07/2019). Health providers lamented that women’s refusal to undertake laboratory tests made it difficult for them to diagnose and to offer clients appropriate medical care (VRF04, observation notes, 30/07/2018). Consequently, ANC managers and workers felt frustrated that they could not provide comprehensive health care to clients.

Health providers perceived that clients did not want to pay for the services, because they did not trust that the money was going to the facilities’ coffers. A midwife stated the following: “*They think that we use the money for ourselves*.” (VRF04, conversation with a healthcare provider, 29/08/2019)

Healthcare managers and workers felt frustrated that sometimes when women were given prescriptions, they bought wrong drugs or expired drugs (VRF02, IDI, ANC Manager; VRF02, IDI, Healthcare Manager). An ANC manager lamented: ‘*You write for them to go and buy [drugs] and when they go, they come back with another drug, so it’s a serious thing* (VRF02, IDI ANC Manager 01). This compromised adherence to treatment.

Some health providers believed that district health directorates had power to assist facilities to acquire adequate drugs and supplies, yet they failed to act accordingly. An ANC Manager expressed her frustration:

‘…*you know the routine drugs (folic acid, iron capsules and a combination of multivite and ferrox) are not available all the time. It’s a challenge, but unless the authority gets up, because we have been complaining and since they [district health directorate] are not on the ground, I don’t know if they don’t feel the pain. But we feel the pains of the pregnant women. You can’t get up and do what you want to do, because they said all initiatives should come from the directorate.’* (VRF02, IDI, ANC Manager)

Two ANC managers in VRF04 perceived that they experienced stock-outs, because their healthcare manager was not supportive. They complained that their facility manager deliberately refused to include the ANC unit’s requisition list in the facility’s general requisition list for drugs and medical consumables. They indicated that they did not trust that their manager was committed to providing resources to facilitate work in the ANC.

Healthcare managers on the other hand blamed the NHIS and the CMS for delaying in reimbursing and supplying facilities with funds and medical products, respectively. They concluded that these two factors accounted for frequent stock-outs and lack of medical consumables in facilities (VRF02, IDI, Healthcare Manager; VRF04, IDI, Healthcare Manager).

## Discussion

This study used ethnographic qualitative methods to explore how healthcare managers resolved resource constraints and financial uncertainties of reimbursement of funds in health care facilities in Ghana. Health facilities experienced frequent stock-outs, delayed reimbursement and had to provide services that they perceived that they would not be reimbursed by government. So they rationed SP, issued prescriptions for women to buy drugs from private pharmacies and introduced partial fees for services and drugs that were previously offered for free. Such managerial decisions influenced utilization of maternal healthcare and IPTp-SP services. Pregnant women who were insured with the NHIS paid half of the cost of MiP services, while those who were uninsured paid the full cost of some of the services that they accessed at the ANC. Poor women who could not afford maternal healthcare delayed ANC visits, skipped ANC appointments or declined to access recommended laboratory tests. Thus, the decision to introduce fees defeated the objective of ensuring equity of access to health care for all pregnant women. Nevertheless, women who could afford the cost of MiP and maternal healthcare remained consistent users. Healthcare providers felt frustrated that sometimes they could not provide comprehensive care to women who could not afford laboratory tests and recommended drugs.

Health facilities passed on some of the hidden cost of maternal healthcare and MiP services such as charges for UPT and G6PD tests to clients, because the NHIS did not reimburse facilities for such services. Some women could not afford such services, which discouraged them from accessing ANC services. Consequently, some did not initiate IPTp-SP early. Late initiation of IPTp-SP can have negative consequences on maternal and neonatal health care in Ghana. This can frustrate the country’s goal of achieving Sustainable Development Goal 3 (SDG 3), ‘*Ensure healthy lives and promote well*-*being for all at all ages’* [77]. Similarly, Klein et al. [78] and Hurley et al. [79] found that facilities charged lump sums for ANC services, which contributed to women’s limited access to IPTp-SP service in Mali. Also, studies conducted in Malawi reported that the introduction of fees for maternal health care resulted in a drop in ANC attendance [57].

Healthcare managers in some of the study facilities introduced fees for some of the maternal health care and MiP services, which were previously free. So, facilities were able to generate IGF to pay creditors, to buy medical consumables (including SP, malaria drugs, RDTs) and to pay casual labourers. Prior to the introduction of the NHIS, health care facilities depended on government subvention and a ‘cash and carry’ system (clients paid fees for health services), to generate funds to undertake such activities [46]. However, in recent times facilities depend on reimbursement from the NHIS for that purpose. The current development suggests that facilities are gradually deviating from the free maternal and malaria care for pregnant women to the previous arrangement of ‘cash and carry’. This has implications for equity in health care delivery, as the poor might not be able to afford maternal health care. Similarly, studies in Ghana and elsewhere found that healthcare managers modified exemptions policies for under 5 year olds and maternal health services, to selective fee payment [37, 44, 46, 80]. The decision helped them to address irregular funding and delays in reimbursement from government. Gross et al. [35], also noted that women had to pay for drugs, cards or diagnostic tests despite the national exemption policy of free health services for pregnant women in South Eastern Tanzania. Navarrete et al. [81] suggested that health care providers were probably unable to execute effectively free health service, because of budgetary constraints and reimbursement uncertainties in Ghana. Such challenges are not limited to Ghana, because similar experiences have been reported in Malawi and Mali [57, 78, 79]. These developments can frustrate Ghana and other sub-Saharan Africa countries’ efforts of achieving universal health coverage.

Women who obtained support from their husbands, family members and friends and those who earned some income utilized comprehensive ANC service. They started ANC early and attended ANC regularly, so they were able to complete at least five doses of SP. They also received appropriate medical care, which contributed to healthy pregnancy and delivery. Other studies have noted that family members and social support groups play an important role in pregnant women accessing ANC and malaria care in Ghana [30, 82]. Other studies have also reported that family members and friends can discourage women from accessing ANC and malaria in pregnancy care [23, 82] Therefore, interventions should target families and communities

Unemployed women, poor women and those who did not receive support from their husbands, family members and friends could not afford maternal healthcare services. Consequently, some failed to attend ANC, others delayed, while some of them skipped scheduled ANC appointments. Such behaviours contributed to some women initiating SP late and others not obtaining the recommended 5 doses of SP. The finding is not surprising, because majority of the 2 percent of women who did not attend ANC in 2017 in Ghana reported that they did not have money [37]. Introducing payment for maternal healthcare and malaria in pregnancy care, can exclude such women, who are among the vulnerable population in Ghana. Ghana’s objective of attaining universal health care coverage can be compromised if the practice continues. Similarly, studies found late initiation and low uptake of IPTp-SP among women who could not afford to pay for ANC and other maternal health services [5, 35, 42, 80]. Also, studies in other parts of sub-Saharan Africa reported that high cost of MiP and maternal healthcare services demotivated women from accessing them [5, 78, 83, 84]. Nevertheless, other studies suggested that limited knowledge on the relevance of IPTp, preference to bring their own cups to fetch water to take the SP drug and worries about side-effects of SP contributed to low uptake [33, 34]. Failure to recognize early signs of pregnancy contributed to late ANC attendance [35] and women with high parity of more than one child visited ANC less often than those with one child [34]. Surprisingly, perceived poor attitudes of health workers contributed to early ANC attendance [35].

Some women combined visiting ANC with self-medicating, using herbal medicines and visiting prayer camps. Women used multiple sources of care in order to cope with increasing cost of MiP and maternal health care. Visiting multiple sources for health care can result in low compliance to medication including anti-malarials, missing scheduled ANC visits and the recommended 5 doses of SP and seeking maternal healthcare late. The current study’s finding has been corroborated by other studies [34, 83]. Rumisha [34] and Hill [83] found that due to high cost of maternal health care, women sought alternative care such as self-medicating, using leftover medicine and using herbal medicine. Another study in Nigeria found that family members and social networks encouraged women to take herbs and to patronize herbal centres [23].

Some women could not pay for comprehensive maternal healthcare and MiP services, so they skipped some aspects of health care such as undertaking laboratory tests and buying and taking prescribed drugs. Consequently, health providers could not make informed clinical decisions in order to offer them appropriate health care. This was a source of frustration and demotivation to healthcare providers, because they could not fully utilize their skills and experiences in the provision of quality maternal health care. In a developing country like Ghana health resources are facing budgetary constraints, so the health care system is stressed. A motivated workforce is crucial for overcoming such an environment. Consequently, it is a matter of urgency that hygiene factors such as medical supplies and consumables that facilitate the provision of health care are readily available [87, 88]. Other studies in Ghana and elsewhere have also reported on health workers losing motivation as a result of lack of health resources to provide optimum care to clients [53, 85–89].

Women in facilities that rationed the SP drug received an average of 3 doses of SP instead of 5, despite some of them starting ANC early and attending ANC regularly. Thus, the decision did not resolve the problem of stock-out of SP. Other studies elsewhere found that women received only one or two doses of IPTp-SP due to shortage of the drug [90, 91]. Gross et al. [92] suggested that health workers could be rationing SP especially the second dose to women, as a way of addressing frequent shortage of the drug. Also, it has been noted that rationing of drugs is not limited to SP and the sub-region. Studies in the USA suggested that rationing of drugs was common, due to reasons such as to restrict access to particular drugs, for profit and to address shortage of a particular drug [92, 93]. However, the practice can deny clients benefits to essential medical products and in extreme cases it can cause harm to clients [92, 93].

Facilities that did not charge fees for SP and other maternal healthcare services, sometimes experienced stock-outs, because they did not have enough IGF to buy medical products. So, women could not obtain all the drugs that were prescribed for them. Healthcare providers found it very difficult to ensure that such women adhered to treatment, since they could not monitor whether they bought prescribed drugs and took them according to the directive given. Consequently, health providers felt frustrated and blamed healthcare managers and district health managers for shortages of drugs and other medical products. Healthcare providers lost trust in healthcare managers and the health care system, which they perceived as not being responsive to their request for medical resources to facilitate provision of quality maternal healthcare. When health workers lose trust in the health care system and in healthcare managers it can have negative consequences on quality healthcare provision [87, 94]. Trust especially between managers and health workers, has been noted to play a crucial role in facilitating effective health care delivery [66, 87, 94].

Clients’ expectation of accessing free and comprehensive maternal healthcare and MiP services was not met, so they lost trust in healthcare providers and the health care system. Instituted charges also contributed to clients distrusting health providers, as some believed that health providers benefitted. This probably contributed to women’s reluctance to pay for maternal health services. It affected adherence to treatment, as some women did not purchase prescribed drugs, so medical conditions that they were diagnosed of were not treated and they believed that health care services were not effective. Therefore, some of the negative outcomes of MiP such as illness, miscarriages and anaemia were given spiritual explanation, which encouraged clients to seek alternative sources of health care. If the trend continues clients could develop more trust in alternative sources of care, which will impact negatively on Ghana’s desire to attain the set targets of millennium development goal 5 [95] and SDG 3 [77]. Trust has been noted to play a crucial role in provider–client relations and adherence to treatment [61, 62, 96, 97]. Tibandebage and Mackintosh [98] and Topp and Chipukuma [99], reported that clients developed distrust of healthcare providers because of they experienced illegal charges in health care facilities. Similarly, Pot et al. [57] found that introduction of user fees compromised the provision of quality of health care and trust relations between pregnant women and health facilities. Losing trust in health facilities can have detrimental consequences on pregnant women’s utilization of maternal health care and IPTp-SP uptake.

## Conclusion and implications

Health care facilities faced challenges in implementing fee-free maternal healthcare and in enforcing IPTp-SP uptake. The challenges included shortage of programme drugs and medical supplies, frequent delays in NHIS reimbursement process and gradual reduction or stoppage in the provision of medical consumables. Healthcare managers exercised power, as stipulated under Act 525, by introducing fees and taking other measures, which they perceived as necessary for ensuring quality service delivery. Yet, their actions conflicted with the fee-free maternal healthcare and MiP policy interventions. The consequences were mixed. Some women could not pay for the services in order to access comprehensive MiP care, which affected their trust in the health care system. However, women who earned some income and others who obtained support from their husbands, relatives and friends were able to afford ANC services. Health workers sometimes felt frustrated that their clients could not afford comprehensive care, because it was challenging for them to provide informed clinical care to such women, so they felt demotivated.

Policy arrangements in the provision of MiP and maternal health resources to facilities appears to be changing over time and managers are devising strategies to cope with this changing trend. The main strategy is passing on some of the cost of maternal and MiP services that used to be free to clients. To be able to find a lasting solution to MiP and Maternal health policy intervention challenges, there is the need for a broader policy response to health facilities’ needs.

The last mile initiative is commendable but it is not enough, because the initiative provides facilities with only drugs. So facilities have to find money to buy consumables, reagents and to pay casual labourers. Healthcare managers have to combine managing and supervising staff with additional burden of finding money to run their facilities, which is stressful. Another important consequence is women are experiencing rising cost and uncertainties in charges on MiP and maternal healthcare services. This affects trusting relations between women and the health care system.

Government and the NHIS need to ensure prompt and continued reimbursement of funds to health facilities and frequent supply of programme drugs and medical supplies to both government and faith-based facilities, which is crucial to effective implementation of maternal healthcare and MiP service delivery. Prompt reimbursement will enable facilities to plan and provide medical supplies for health service delivery early. This will contribute to quality health care provision and advance the goal of achieving the reset Abuja targets for IPTp-SP in Ghana.

Also, effective and regular communication between health facilities and agencies responsible for providing essential medical resources for health care delivery is crucial. Effective communication will give facilities the forum to communicate implementation challenges to the appropriate authorities, so that lasting solutions could be explored.

Community engagement is critical. Health care facilities need to engage communities to help them appreciate the challenges and demands of maternal healthcare and MiP care. Facilities also need to embark on regular mass education campaigns on maternal healthcare and MiP care in communities that utilize their services. Such education will increase community awareness on the benefits of ANC attendance and the effects of malaria in pregnancy. This could facilitate communal support both economic and social for poor women to access ANC service, which can facilitate IPTp-SP uptake.

Managers need to find innovative ways of raising funds through community groups, district agencies such as the district assembly and non-governmental organizations, to support funding gaps in maternal healthcare and MiP care. This is because some communities are well off and could help health facilities to meet some of their needs.

## Limitation of the study

The methodological choice of ethnography in five districts over a period of 12 months, had the possibility of researchers going’native’, that is becoming part of the community and losing their role as neutral observers. To overcome such bias, the team held weekly conference calls for all the researchers working in different facilities and communities, to discuss experiences and to ensure that team members did not deviate from the objectives of the study. Ethnographers argue that both the researcher and the research participants are vested with power. Hence, both parties can be selective with the information that they choose to provide, which can result in study bias [100–102]. To ensure that researchers and research participants were not selective or bias in the information that they chose to present, this current study used multiple data collection methods. The researchers who were ‘outsiders’ (not community members and healthcare implementers) in the study field, had the tendency to misinterpret and misrepresent study participants’ experiences. This bias was minimized by the researchers first conducting an ethnographic immersion into the study field and staying in the study field for long periods of time in order to become familiar with community practices. Also, the researchers conducted follow-up visits to study participants to clarify experiences that did not appear obvious to researchers during analysis of the first set of data. Additionally, a neutral researcher was hired to support coding. The neutral researcher and a member of the research team coded the data independently and compared the outcome to ensure neutrality. Nonetheless, to be able to understand multiple levels of behaviour and linkages between healthcare decisions and behaviour, conducting ethnography over a long period of time was the most appropriate study design. Also, adopting grounded theory approach enabled the study to probe complex phenomena in order to represent the different actors that this study sought to understand.

## Supplementary information

**Additional file 1.** MiP intervention study_Managers Interview Guide.

**Additional file 2.** MiP intervention study_Supplementary HF Observation CL.

**Additional file 3.** MiP Intervention study_IDI guide_Health Providers.

**Additional file 4.** MiP Intervention study_Community observation CL.

**Additional file 5.** MiP intervention study_HF Observation CL.

**Additional file 6.** MiP intervention study _IDI guide_Pregnant women.

## Data Availability

The datasets used and/or analyzed for this study are available from the corresponding author’s institution on reasonable request.

## Footnotes

1 In the year 2000, African leaders met in Abuja under the Roll Back malaria Partnership and set targets of 60% of pregnant women having access to anti-malarial chemoprophylaxis or intermittent preventive treatment (IPTp) and 80% of women using insecticide-treated nets by 2005. More ambitious targets were reset to 100% of women utilizing both interventions by 2015 [13].

2 Conversation is defined as: ‘*An informal conversational interview. An unplanned and unanticipated interaction between an interviewer and a respondent that occurs naturally during the course of fieldwork observation. It is the most open-ended form of interviewing* [103].

3 MA initially trained the RAs on immersing themselves in health facilities and study communities, conducting transect walks and non-participant observation and taking field notes, Other activities were conducting in depth interviews and conversations. The research assistants participated in two other training sessions during the data collection phase.

4 An urban and a rural district were selected in each region in order to reflect diverse cultural experiences. The initial plan was to select a rural and an urban district in each region, however upon our visit to the Ashanti region, we realized that one of the districts had recently been divided into two, so we went ahead to work in the two districts.

5 While written consent is recommended for study participants, verbal consent can be used in situations where time is of the essence [104]. In this study women who were attending ANC were invited to participate in conversations and in depth interviews. Those who had ample time for an interview were given time to reflect and to consent. They were followed to their homes at a later date for interviews. However, RAs conversed with women who were willing to participate in the study, but did not have enough time to participant in IDIs. Such study participants granted oral consent to participate in the study.
