# Supplementary material for: Managing intermittent preventive treatment of malaria in pregnancy challenges: an ethnographic study of two Ghanaian administrative regions
Source: Malar J. 2020 Sep 25;19:347. doi: 10.1186/s12936-020-03422-2 (PMC7519547; doi:10.1186/s12936-020-03422-2)
Supplement: Supplementary file 1 — Additional file 1. MiP intervention study_Managers Interview Guide. [file 12936_2020_3422_MOESM1_ESM.docx]

**MALARIA RESEARCH CAPACITY DEVELOPMENT FOR WEST AND CENTRAL AFRICA: (MARCAD)**

**Ethnographic study on health system, interpersonal, socio-cultural, environmental and community factors influencing uptake of preventive measures and management of malaria among pregnant women in Ghana**

**Supplementary Observation checklist in health facilities 1**

**Date: 24^th^ July, 2018**

**Bednet**

Observe how it is given out to pregnant women.

The message the pregnant women are given on how to use it.

**IPTp**

Observe ANC consultation. How nurse provides ANC services

How SP is given

What message is the pregnant woman given before being given SP?

What message is the pregnant woman given after taking the SP?

**Malaria cases**

Observe how women with malaria present at the facility.

Observe how the nurse goes about with treatment of MiP.

Whether woman is treated or asked to go for test or undergoes RDT

After RDT or lab test what happens: how is treatment given, what is the pregnant woman told by the nurse?

How is the pregnant woman treated at the lab?

How is the pregnant woman treated at the pharmacy?

**Admission/detained**

Where women are detained or admitted for malaria, find out at what stage that women are admitted

If women are able to hold a discussion find out how they got malaria

If unable to talk take their contact and after they are discharged visit their homes to find out how they got malaria. But note those allergic to SP.

Table for observation

Consulting room no.

Date:

| Client no. | IPTp given under DOT | Given without DOT | IPTp Not given | Comment (Education given or not before and after IPTp) |
| --- | --- | --- | --- | --- |
|  |  |  |  |  |
|  |  |  |  |  |
|  |  |  |  |  |
|  |  |  |  |  |
|  |  |  |  |  |
|  |  |  |  |  |
|  |  |  |  |  |
|  |  |  |  |  |

Bednet

| Client no. | Given bednet | Comment (Education given or not before and after issuance) |
| --- | --- | --- |
|  |  |  |
|  |  |  |
|  |  |  |
|  |  |  |
|  |  |  |
|  |  |  |
|  |  |  |
|  |  |  |

Malaria treatment

| Client no. | Test conducted | How treatment is provided | Comment (interaction with nurse, education given etc) |
| --- | --- | --- | --- |
|  |  |  |  |
|  |  |  |  |
|  |  |  |  |
|  |  |  |  |
|  |  |  |  |
|  |  |  |  |
|  |  |  |  |
|  |  |  |  |
