# Supplementary material for: Managing intermittent preventive treatment of malaria in pregnancy challenges: an ethnographic study of two Ghanaian administrative regions
Source: Malar J. 2020 Sep 25;19:347. doi: 10.1186/s12936-020-03422-2 (PMC7519547; doi:10.1186/s12936-020-03422-2)
Supplement: Supplementary file 2 — Additional file 2. MiP intervention study_Supplementary HF Observation CL. [file 12936_2020_3422_MOESM2_ESM.docx]

**MALARIA RESEARCH CAPACITY DEVELOPMENT FOR WEST AND CENTRAL AFRICA: (MARCAD)**

**Ethnographic study on health system, interpersonal, socio-cultural, environmental and community factors influencing uptake of preventive measures and management of malaria among pregnant women in Ghana**

**IDI guide for in-charges, medical superintendents/directors**

**Date: 19^th^ November, 2018**

**Name of facility:**

**Domain 1: Background information**

**I will appreciate it very much if you would kindly tell me about yourself:**

- 1. **Name:**
  2. Educational background?
  3. Designation? Qualification
  4. How long you have been a manager (mention his/her designation)?
  5. Number of years in current designation
  6. **Age (not compulsory)**
  7. What is the catchment population that you offer services to? (Communities that utilize the services of the facility, it can also refer to the CHPS zones and other facilities that refer cases to the facility)

**Domain 2: Maternal Health Policies and Maternal health care**

1. Please which are the existing national policies on maternal health care that are being implemented in this facility?
2. Which are the recommended ANC policies that are being implemented in this facility?
3. How equipped is your facility (work force, supplies - bed nets, equipment, reagents etc. all the non-consumables etc., infrastructure, medicines) in the implementation of the ANC policies that you have mentioned?
   - Do you have adequate staff to run maternity services? (maternity, ANC, labs, pharmacy)
   - How do you organize service delivery to cater for any gaps that you have on workforce, especially for maternity services?
   - How often do your staff who provide maternity services receive training?

***Let’s now focus on malaria infection in pregnancy***

1. Kindly educate me on the policies concerning prevention and treatment of malaria in pregnancy that are being implemented in this facility (testing and treating; preventing malaria in pregnancy - ITNS and IPTp-SP).
   - What are the challenges in implementing these interventions in this facility?
   - How do you address these challenges (refer to each of the challenges that interviewer mentioned)
   - What factors promote implementation of these interventions in this facility?

**Domain 3: Supplies, medicines and equipment**

1. Which aspects of the maternity services that you provide are covered by the fee-free delivery policy?
2. Which aspects of maternity services are not covered by the fee-free delivery policy?
   - Probe the following if not mentioned as covered or not by the fee-free delivery policy: cost of testing and treating of malaria in pregnancy, bed net and SP.
3. Who or which body is responsible for providing you with your medical supplies especially malaria prevention, testing and treating (routine drugs, SP, bed net, lab tests, and treatment drugs for malaria) for pregnant women? (Probe government - medical stores, district health directorate/regional health directorate, NHIS, etc.)
4. How satisfied are you with (ask for each body mentioned eg government, NHIS etc.) the response to your needs for medical supplies? (Probe why)
5. What about medicines (maternal health drugs: the free ones and those that have to be paid for), do you receive adequate quantities for health delivery? (If No, why?)
6. What about equipment? What are the challenges that you face with regards to your need for medical equipment (laboratory and others used for testing for malaria in pregnancy)?
7. How does lack of medicines, supplies, and equipment affect ANC provision in your facility?
8. Do you experience stock-outs (routine drugs, SP, bed nets, malaria treatment drugs, reagents for lab etc. ask for each)?
   - **If no**, ask what arrangement has been made that ensures that they do not experience stock-outs.
   - **If yes**, ask why that happens. Ask how they cope with it and how they have strategized to ensure that they have equipment and supplies in place, in order to be able to deliver maternal health services without any interruption
   - How do you deal with challenges arising from **fee-free delivery services** to ensure that maternal health service provision is not disrupted by the following challenges: reimbursement, limited staff numbers, lack of equipment, supplies etc.
9. I have observed that the insured clients pay for some of the items that are supposed to be free for pregnant women, may I know the reasons? (**Mention those that you observed to be peculiar to the manager’s facility and discuss**)
10. How beneficial is the government’s role in supporting fee-free delivery care to health facilities?
11. How does the NHIS’ role as the agency responsible for implementing the government’s fee-free maternity services facilitate maternal health service delivery in your facility?
    - Tell me about the arrangements you have with the NHIS!
12. Do you have other institutions supporting you? If yes, please mention them. (Sometimes international bodies such as USAID, NGOs, individuals, church organizations etcetera provide support such as drugs, equipment to health facilities)
13. Tell me about the requisition process and arrangements in this facility.
    - How does the ANC get its supplies such as SP, folic acid, fesolate, multivite?
    - Ask about arrangements for laboratory and pharmacy services

**Domain 4: Client care and staff attitudes**

1. What are some of the factors that hinder/prevent pregnant women from seeking ANC care in your facility?
2. What factors encourage pregnant women to use your services?

**Domain 5: Recommendations**

1. What must the government do in order to ensure that you have the right equipment and supplies to provide appropriate ANC services?
2. How can the government enhance your capacity to provide malaria interventions?
3. What can the NHIS do to enhance your capacity to provide maternal health care services? What about the NHIS’ role in ensuring that you are able to deal with malaria in pregnancy?
4. What do you think you could do as a facility to improve maternal health service delivery?
5. How can your facility improve prevention and treatment services for malaria in pregnancy?
