# Supplementary material for: Managing intermittent preventive treatment of malaria in pregnancy challenges: an ethnographic study of two Ghanaian administrative regions
Source: Malar J. 2020 Sep 25;19:347. doi: 10.1186/s12936-020-03422-2 (PMC7519547; doi:10.1186/s12936-020-03422-2)
Supplement: Supplementary file 3 — Additional file 3. MiP Intervention study_IDI guide_Health Providers. [file 12936_2020_3422_MOESM3_ESM.docx]

**MALARIA RESEARCH CAPACITY DEVELOPMENT FOR WEST AND CENTRAL AFRICA: (MARCAD)**

**Ethnographic study on health system, interpersonal, socio-cultural, environmental and community factors influencing uptake of preventive measures and management of malaria among pregnant women in Ghana**

**Interview guide for healthcare providers**

**DATE: 26^th^ June, 2018**

**Background information**

Facility name

Name of health worker

Age

Professional background

Number of years of training

Number of years you have been working in the facility

**Malaria case management**

1. Tell me about your work and experience as a nurse/midwife/physician assistant/lab technician.
2. What are the ailments that pregnant women visit this facility to seek healthcare on? (probe for malaria)
3. How common is malaria among pregnant women who visit this facility?
4. Kindly explain to me the policy on treating malaria in pregnancy.
5. How are such cases managed?
   1. Probe whether lab test or RDT is done or both are done.
   2. Treatment provided (if treatment is provided without test, ask why)
   3. The education given to the women afterwards etc.)
6. Kindly tell me about your experience on pregnant women’s adherence to malaria treatment
7. Are there instances that a pregnant woman can visit this facility to seek treatment for malaria several times before delivery? If no/yes, why is that the case?
8. What are the primary intervention strategies that you use to prevent malaria in pregnancy? (Probe for ITNs, IPTp-SP)

**BED NET**

1. What is the existing policy on insecticide treated bed nets (ITNs) in Ghana?
2. Please educate me on distribution, availability and supply.
3. How do the pregnant women who visit this facility get bed nets for use? (Probe: Are the nets given free of charge? If yes why? If no why?)
4. Do you give each pregnant woman a net? (If yes, why do you give a bed net to each pregnant woman/if no why not?)
5. Can you tell me when you usually give out bed nets to pregnant women?

(Probe at what point/stage of pregnancy that bed nets are given out)

1. What messages do you give to them when you distribute the bed nets to them?
2. How do you ensure that pregnant women sleep under the bed nets that you give to them? (Probe: how can you tell whether a pregnant woman uses the bed net that you gave to her?)
3. What are the alternative uses of bed nets in the communities that access your services?

**IPTp**

1. What is the policy on IPTp-SP?
   1. When should pregnant women start taking sulfadoxine-pyrimethamine (SP)?
2. How is SP supposed to be given (explore for direct observation therapy (DOT). If no DOT, probe why and how they still ensure that when the pregnant women take it home they still take it)
   1. Cost of it, whether payment is made or not.
3. How many times are they required to take it?
4. When should they take the last dose?
5. Do you have SP throughout the year? (Probe for stock-outs. If yes/no, probe why)
6. Do you provide them with water to take it? If no, where do they get water to take it? If the woman does not have water, what do you do? **Observe whether nurse has water or not and how ANC attendees behave with SP.**
7. What explanation do you usually give to women to convince them to take SP?
8. Do you have cases of women refusing to take SP? Probe: how do you address such cases?
9. What do you tell them in connection with retuning for a repeat dose? (Probe for first timers, second timers etc.) **Observe at ANC.**
10. What is the uptake of IPTp in this facility? How often do women return to take a second dose? What about the third dose, etc.? **(Take data on IPTp 1, IPTp2 etc from records: at a later date return to discuss why the different in IPTp coverage for 1, 2,3,4,5 etc.).** What factors do you think account for the gaps in the uptake of IPTp? How can these gaps be resolved?
11. On average how many doses does a pregnant woman who visits this facility take before delivery?
12. When is usually the last time they take SP before delivery?
13. What factors facilitate uptake?
14. What plans do you have to promote increase uptake of IPTp?

**ANC**

1. At what stage of pregnancy do pregnant women begin to attend ANC in this community? (probe why they come at that stage)
2. How frequently do they come for ANC before delivery?
3. What messages are they given when they come for ANC (Message on relevance of visiting frequently, drugs to take, etc.)
4. How is health education done (is it Focused, or general talks)? **Take time to observe later on**
5. What is the content of the health education? **(Also visit to observe)**
6. **Observe payment for services and how the National Health Insurance Scheme works in the facility.**

**Other strategies aimed at preventing malaria in pregnancy**

1. Can you tell me what preventive strategies that community members use to prevent malaria apart from visiting the hospital?
2. What about pregnant women, besides this facility, which are the other places that they visit to seek treatment for malaria related ailments?
   1. What about prevention of malaria ailments?
3. What about herbs, spiritualist, churches, prayer camps etcetera (Ask of each of them separately).
4. What influence do mother in-laws have on pregnant women’s use of health services? (Probe for husbands, father in-laws, friends etc.)

**Other issues on malaria treatment**

1. How will you assess the current policy for prevention and case management of malaria in pregnancy?
2. Do you think we give pregnant women too much drugs during pregnancy? (Let respondent explain his/her response).
3. What do you think are the best ways to treat malaria in pregnancy?

**Recommendations**

1. What are your recommendations towards improving prevention of malaria in pregnancy?
2. What are your recommendations towards improving treatment of malaria in pregnancy?

**If you have any other suggestions or recommendations that you think could be useful on the subjects that we have discussed, we would be happy to listen to them.**

**Many thanks for granting me this interview.**
