# Supplementary material for: Managing intermittent preventive treatment of malaria in pregnancy challenges: an ethnographic study of two Ghanaian administrative regions
Source: Malar J. 2020 Sep 25;19:347. doi: 10.1186/s12936-020-03422-2 (PMC7519547; doi:10.1186/s12936-020-03422-2)
Supplement: Supplementary file 4 — Additional file 4. MiP Intervention study_Community observation CL. [file 12936_2020_3422_MOESM4_ESM.docx]

**MALARIA RESEARCH CAPACITY DEVELOPMENT FOR WEST AND CENTRAL AFRICA: (MARCAD)**

**Ethnographic study on health system, interpersonal, socio-cultural, environmental and community factors influencing uptake of preventive measures and management of malaria among pregnant women in Ghana**

**Observation Checklist**

**2^nd^ April, 2018**

1. Bed net
2. IPTp
3. Indoor spraying
4. Management of malaria in pregnancy using traditional and orthodox

**Environmental**

- Design of houses and rooms: describe architecture
- Location and distance to health facility
- Pattern of settlement
- Sleeping arrangements
- Surrounding bushes
- Drainage system
- How long people have to travel to engage in activities (livelihoods etcetera)

**Socio-cultural**

- History of community: Ethnic origins
- What are the existing kin relations
- Age structure of the community
- Socio-economic status of the households
- Livelihood of individuals and community (men, women, etc.)
- Household structure and relations
- Decision making structure and process
- Myths and beliefs
- Religious affiliation
- Educational status

**Community**

- What are the activities that people engage in?
- Where do people hang out (men, women, age categories, etcetera)
- How people interact
- How community activities are organized
- What kinds of entertainment and other opportunities exist
- What is the source of information on health care
- How often are community members given health talks?
- What are the contents of the messages?
- Which people provide health talks?
- Which category of people attend the talks?
- What are the health concerns of the community members?
- How long do people stay out before going to bed?

**Health system factors**

- Which health facilities do community members prefer to go to?
- What are the reasons that lead community members to the health facility?
- What does ANC mean to community members, households and to the pregnant woman?
- At what stage of pregnancy do women in the community begin to go for ANC?
- Why do women go for ANC?
- What are their expectations?
- What does IPTp mean to the women and their families?
- What are their perceptions about the healthcare system?
- What are their perceptions about health workers?

**Pregnancy**

- How is pregnancy perceived in the community?
- What are the myths and misconceptions about pregnancy?
- What are the terms given to pregnancy?
- What are the ailments that afflict pregnant women?
- Ho are such ailments treated?
- What are the beliefs about taking drugs during pregnancy?
- What causes miscarriages?
- What does low birth weight mean?
- What about premature delivery?
- Mortality in pregnancy?
- What is the culture of pregnancy inception, is it announced?
- How often do pregnant women go to the health facility?
- Why do they go to the health facility?
- What is the role of traditional birth attendants?
- What is the role of community based volunteers?

**Chemist shops**

- Where are they sited?
- What do they do for the community members?
- Which category of community members visit them?
- Drug peddlers

**Traditional healers**

- Where are they located?
- What ailments do they treat?
- What are their roles in pregnant women’s lives?
- How often do people visit them
- Which categories of people visit them?
- Fetish priest/priestess
- Herbalist

**Religion**

- What religious denominations exist?
- Where do the community members worship?
- How does religion influence what community members do and say?
- What is the role of pastors in health care?

**Health seeking behavior**

- What is the culture of seeking health care?
- Which is the first point of call when people fall ill?
- What about pregnant women, where do they prefer to seek help?

**Indoor spraying**:

- Awareness?
- Where?
- How is it done?
- How often it is done
- Which houses practice it

**Mosquito repellents**

- Which houses use them
- How often are they used?
- Why are they used (multiple factors, mosquitoes, other insects)

**Cream**

**Bednet**

- How many people are in a household?
- How many nets does the household owe?
- What is the sleeping pattern?
- How many persons in a household share a bednet?
- How are the bed nets managed: washing, age, appearance: new or old-describe?
- Aside the bed net, what other things does the household use to prevent mosquito bites? Probe on the use of leaves and other local substances?
- How are the nets hanged in the room?
- Who is responsible for hanging the nets in a room?
