# Supplementary material for: Managing intermittent preventive treatment of malaria in pregnancy challenges: an ethnographic study of two Ghanaian administrative regions
Source: Malar J. 2020 Sep 25;19:347. doi: 10.1186/s12936-020-03422-2 (PMC7519547; doi:10.1186/s12936-020-03422-2)
Supplement: Supplementary file 5 — Additional file 5. MiP intervention study_HF Observation CL. [file 12936_2020_3422_MOESM5_ESM.docx]

**MALARIA RESEARCH CAPACITY DEVELOPMENT FOR WEST AND CENTRAL AFRICA: (MARCAD)**

**Ethnographic study on health system, interpersonal, socio-cultural, environmental and community factors influencing uptake of preventive measures and management of malaria among pregnant women in Ghana**

**Checklist for health facility observation**

**Draft date: 20^th^ June, 2018**

**Compliment observation with conversations to clarify and explain for a comprehensive understanding of observations made.**

**Observation should focus on ANC OPD, if there is no ANC OPD then observe from general OPD. Other areas to observe are the maternity/O&G ward (pregnant women who come on admission-check malaria related admissions) pharmacy, laboratory, etc.**

**Environment**

- The location of the facility (distance from the major settlements)
- The catchment area that it serves
- Describe the main structure of the facility (include how each specialized area is designed and connected to service delivery)
- In Ghana health service what is the level, include kinds of services that are provided in the facility.
- How health care is organized
- Allocation of roles
- Number of hours service is provided
- How is maternal health care organized?

**Management of malaria in pregnancy**

- What ailments do pregnant women present at the facility?
- What percentage of the cases are malaria cases?
- How are malaria cases addressed by health providers?
- Check for RDT and lab test done before treatment
- Check if RDT/LAB test are not done but treatment is given, find out why
- How is treatment after test or no test administered to pregnant woman?
- How is the interaction between health workers and clients (please describe)
- Check the frequency of pregnant women coming down with malaria
- Converse with the women to find out how they protect themselves from malaria (whether they use net at home, come for SP, use other forms of protection such as repellents, spray, coil, traditional birth attendants, herbalists, prayers etc.)

**Health system factors**

- Which health facilities do community members prefer to go to?
- What are the reasons that lead community members to the health facility?
- What does ANC mean to community members, households and to the pregnant woman?
- At what stage of pregnancy do women in the community begin to go for ANC?
- Why do women go for ANC?
- What are the expectations?
- What does IPTp mean to the women and their families?
- What are the perceptions about health care system?
- What are the perceptions about health workers?

**ANC attendance**

- What kind of ANC is provided in the facility (Focused etc.)
- How frequently and times that ANC services are provided
- Observe the components of actual provision. (Health talk-content, checking of BP, sugar in urine, etc.)
- What is the package given at ANC?
- Which category of women come for ANC? Categorize women into 1^st^, (1-3 months) 2^nd^ (4-6 months) and 3^rd^ (7-9 months) trimesters. Also include socio-cultural background, age, distance from home to facility
- At what stage of pregnancy do they come for ANC?
- What is their expectation at ANC?
- How frequently do they attend ANC?

**Hospital Records**

Go through the hospital records to find out the number of malaria cases for 2015, 2016, 2017 and 2018 June ending.

| **Suspected malaria cases** | **2015** | **2016** | **2017** | **June 2018** |
| --- | --- | --- | --- | --- |
| **Suspected malaria cases tested** |  |  |  |  |
| **Tested Positive malaria cases** |  |  |  |  |
| **Not tested but treated** |  |  |  |  |
| **Malaria in pregnancy** |  |  |  |  |

**ANC records**

Also take records of IPTp coverage from 2013-2018 (tabulate it just as done above)
