# Supplementary material for: Managing intermittent preventive treatment of malaria in pregnancy challenges: an ethnographic study of two Ghanaian administrative regions
Source: Malar J. 2020 Sep 25;19:347. doi: 10.1186/s12936-020-03422-2 (PMC7519547; doi:10.1186/s12936-020-03422-2)
Supplement: Supplementary file 6 — Additional file 6. MiP intervention study _IDI guide_Pregnant women. [file 12936_2020_3422_MOESM6_ESM.docx]

**MALARIA RESEARCH CAPACITY DEVELOPMENT FOR WEST AND CENTRAL AFRICA: (MARCAD)**

**Ethnographic study on health system, interpersonal, socio-cultural, environmental and community factors influencing uptake of preventive measures and management of malaria among pregnant women in Ghana**

**Study guide for pregnant women**

**Version dated 3^rd^ June, 2018**

**Background characteristics**

Name

Age

Community coming from

Educational level

Occupation

**Section 1: Knowledge on Malaria**

**In this section I would like us to discuss your knowledge on malaria**

1. What are the ailments that commonly afflict people in your community? (Probe for all including malaria)
2. How common is malaria infection in your community?
3. How often do people get malaria?
4. How do people perceive malaria in your community?
5. How common is malaria among pregnant women?
6. Can you tell me how a pregnant woman can get malaria?
7. How do you know when you are afflicted with malaria during pregnancy?
8. What are the effects of malaria in an infected person?
9. What are the effects of malaria in a pregnant woman? (Probe effect on mother, Probe effect in unborn child)
10. What are the things that pregnant women can do to prevent themselves from getting malaria during pregnancy?

**Section 2a: Knowledge, attitude and use of bed nets**

**I would like us to talk about your knowledge and practice in regards to bed nets**

1. Okay, let us now talk about mosquito nets (ITNs), do you have a mosquito net? (If no, skip to section 2b)
2. How did you get it?
3. Who encouraged you to acquire a bed net?
4. Can you describe to me how you dealt with your net on the first day that you had it?
5. Do you sleep under the bed/mosquito net? If yes, how often/If no, why not?
6. When did you start sleeping under the bed net? *When was the last time that you slept under a bed net? *Did you sleep under the bed net last night?
7. What are your reasons for sleeping under a mosquito net?
8. What times do you sleep under the bed net?
9. (Calculate the number of hours the respondent is not sleeping under a bed net). What do you do to prevent mosquito bites during the hours that you are not sleeping under the bed net?
10. Can you describe to me your net using behaviour before you became pregnant?
11. Has there been changes in how you use the bed net since you became pregnant? If yes what are the changes, if no, why not?
12. Are there specific instances that you don’t feel like using the net? Give examples
    1. Do you sleep under a bed net during hot weather?
    2. What about during the cold weather? (Probe why if yes or no for both seasons)
13. Is the net hanged on the bed all the time? Who is responsible for hanging your net? Can you hang it yourself?
14. What do you do to the net when you wake up?
15. Who influences you to sleep under a bed net?
16. What would people in your household say if you do not sleep under a bed net?
17. Kindly tell me what you do to your net when you think that it is looking dirty?
    1. Probe to a. calculate the number of times the net is washed in a year b. How it is washed
18. What about when it gets torn?

**Section 2b: Family and community bed net use**

**I would like us to discuss knowledge and use of bed net in the community**

1. Which people use bed nets in this community?
2. How did the use of bed nets come about in this community?
3. How many are you in your household?
4. How many nets do you have in the household?
5. What is the sleeping pattern?
6. How many of you share a bed net?
7. What are the other uses for bed nets in this community?

**Section 2c: Other preventive measures**

**Let’s talk about other preventive measures for malaria**

1. Aside the bed net, what other things do you do to prevent mosquito bites?
   1. Probe on the use of IRS, mosquito repellents, leaves of plants and other local substances. For each mentioned ask subsequent questions etc.
2. How protective is mentioned source?
3. Where did you learn to use it?
4. How did/do you usually get it?
5. How common is its use in the community
6. Which category of people use it?
7. Why do they use it?

**Section 3a: ANC**

**We are going to talk about issues on ANC, the drugs you are given at ANC and how you are treated at ANC**

1. At what stage of pregnancy do women in this community start attending ANC?
   1. If respondent reports late/early start of ANC probe: factors that prevent/encourage her from attending ANC? Probe on social, economic, distance, and time factors.
2. When did you start attending ANC?
   1. Probe for reason for starting at that period, the stage of pregnancy, the number of times she has attended ANC since becoming pregnant etc.
3. What are the factors that influence you to continue to attend ANC?
   1. Probe for social, economic, distance, and time factors and how they affect respondent’s ANC attendance
4. Who decided that you should start attending ANC?
   1. Probe why that person had to decide?
   2. Based on whose prompting/influence do you continue to attend ANC?
5. Tell me about the cost you incur when you attend ANC.
6. How do you raise money for that?
   1. If she mentions a financier, ask how the financier influences her decision to attend ANC.

**Now let us talk about what you are given at the ANC and how you are treated**

**Section 3b: IPTp intervention**

1. When you go for ANC what are some of the medications that you are given? (Let her describe them and ask why she is given each of the drugs that she mentioned).
2. She should narrate how it is taken if she mentions and describes SP (DOT or not, probe)
3. Why are pregnant women given some medicines when they go for ANC?
   1. Probe for sulfadoxine-pyrimethamine (SP). Probe for the benefits of SP to mother, probe for benefits to unborn child as well. Also probe for the risks involved in taking SP.
4. How many times have you been given SP at the health facility? (If non, ask why)
5. How many times do you think you need to take it during your pregnancy?
6. What are you usually told by the nurse before you take the SP?
   1. Also ask what she is told by the nurse afterwards
7. What does she feel about the drug and what are her future intentions of taking or not taking the drug during the period that she is pregnant?
8. What have you been told about taking drugs during pregnancy?
9. What are your experiences in taking drugs whiles pregnant?

**Section 3c: Health system factors**

1. Can you share examples of experiences you encountered when you attend ANC?
2. Kindly tell me how you are treated by (midwives/ doctors/laboratory technicians) when you go for ANC. Probe on attitude of health workers. (Probe on attitude (friendliness, communication of health workers, medical treatment given (consultation, medication and health education, waiting time, out of pocket payment.)
3. Are you given education on malaria? What can you remember/please tell me something about it.
4. Why do you choose to attend ANC in this facility?
5. What do you like about the services you receive when you attend ANC?
6. What don’t you like about the services that you receive at the ANC?
7. Can you share examples of experiences that you had when you attended ANC?
8. Besides attending ANC, have there been occasions that you visited the hospital to seek health care during pregnancy?
9. Ask when, which facility and what was the ailment? Probe for malaria. If malaria is mentioned ask the subsequent questions:
10. Why she decided to visit the health facility to seek health care
11. How it was managed by the health officials such as the kind of tests that were conducted.
12. The kind of treatment that was given and how she adhered to the treatment regimen.

**Section 4a: Malaria case management at individual and household level**

1. Where do people usually seek health care when they are ill? Probe on Self-medication, Orthodox, religious and herbal treatment options.
2. What informs the decision to seek health care from the mentioned source? (Probe: Which do you prefer and why?)
3. Where do pregnant women usually seek healthcare when they are ill? (Probe on the use of alternative sources, local remedies and medicines by pregnant women)
4. When you suspect that you have malaria what are the steps that you take to ensure that you obtain treatment?
5. Besides the hospital, which are the other ways or places that you seek treatment when you have malaria? (Probe on self-medication, health facility, herbalist, church, Traditional birth attendant, community health worker, prayer camp etc.)
6. Why do you go to the mentioned place for treatment? (Pick all places mentioned and ask)
7. Who in the household decides on where you should go to?
8. Who provides money for treatment?

**Section 4b: Local and traditional remedies to preventing and managing malaria**

1. Tell me more about any herbal or traditional medications used to prevent malaria and other parasitic infections.
2. Why do you go for the remedies mentioned?
3. Can you describe to me how (each of) these remedies are prepared?
4. What is the source of these remedies? Who goes to fetch them?
5. On a scale of one to ten how many pregnant women in this community take them?
6. What about you, do you take them? (If yes/no, probe why)

**Many thanks for granting me this interview. I will follow up with you in case i am not able to understand some of the responses that you gave me.**
